# Role of occupational health in managing non-communicable diseases in Brunei Darussalam

**DOI:** 10.3402/gha.v7.25594

**Published:** 2014-11-06

**Authors:** Pg Khalifah Pg Ismail, David Koh

**Affiliations:** 1 Department of Health Services, Ministry of Health, Bandar Seri Begawan, Brunei Darussalam; 2 Occupational Health and Medicine, Universiti Brunei Darussalam, Bandar Seri Begawan, Brunei Darussalam; 3 SSH School of Public Health, National University of Singapore, Singapore

**Keywords:** non-communicable diseases, occupational health

## Abstract

Like most ASEAN countries, Brunei faces an epidemic of non-communicable diseases. To deal with the complexity of NCDs prevention, all perspectives - be it social, familial or occupational – need to be considered. In Brunei Darussalam, occupational health services (OHS) offered by its Ministry of Health, among others, provide screening and management of NCDs at various points of service. The OHS does not only issue fitness to work certificates, but is a significant partner in co-managing patients’ health conditions, with the advantage of further management at the workplace. Holistic approach of NCD management in the occupational setting is strengthened with both employer and employee education and participation, targeting several approaches including risk management and advocating healthy lifestyles as part of a healthy workplace programme.

Brunei Darussalam, with a population of over 400,000, currently faces an increasing burden of non-communicable diseases (NCDs) such as diabetes, heart disease, and cancer. NCDs account for over half of all deaths locally (1), with the four leading causes being cancer, heart diseases, diabetes mellitus, and cerebrovascular diseases. This is similar to other Southeast Asian countries (2), where chronic NCDs account for 60% of deaths. The underlying causes for this include smoking, unhealthy diets, and inadequate physical activity. Preliminary results of a recent national survey on health and nutrition (3) showed the number of obese adults almost doubling since the last study in 1998.

As a Member Country of the Association of South East Asian Nations (ASEAN) and World Health Organization (WHO), Brunei Darussalam actively participates and contributes in WHO and ASEAN health-related meetings. The country's commitment to tackle NCDs was reflected when the Bandar Seri Begawan Declaration on NCDs in ASEAN was successfully adopted during the 23rd ASEAN Summit in 2013 (4). His Majesty the Sultan of Brunei Darussalam has repeatedly stressed the importance of the nation's health, including the impact of NCDs and called for sustainable actions in adhering to healthier lifestyles in tackling NCDs^1^

To deal with the complexity of NCDs, all perspectives – be it social, familial, or occupational – need to be considered. NCD prevention and control need a holistic, integrated, and multilevel approach requiring strong and sustained commitment and actions from all sectors to address NCDs. Within the working population, work and workplace risk factors can cause or exacerbate NCDs.

With this in mind, occupational health services (OHS) offered by the Brunei Darussalam's Ministry of Health, among others, provide screening and management of NCDs at various points of service – particularly at entry and at periodic intervals for specific jobs as well as walk-in clinics and referrals from other specialties. These clinical activities complement other essential OHS activities such as workplace surveillance, workplace health advice and promotion, as well as workmen's compensation issues. Thus, a holistic approach to management of workers’ health status, workplace environment as well as policy change can be implemented.

This approach is in line with WHO recommendations of workplaces as healthy settings to promote health, including interventions for NCDs. It is also in accord with the WHO Global Plan of Action on Workers Health 2008–2017, which was adopted at the 60th World Health Assembly (5). Risk factors and hazards at the workplace – for example, sedentary work, work stress, exposure to carcinogens, shift work, unsupportive environments, such as provision of unhealthy food at workplaces – which can contribute to NCDs can be effectively addressed through these initiatives.

OHS in Brunei Darussalam is still relatively new as compared to other well-established health services such as community health. Workers in Brunei Darussalam are exposed to a plethora of risks and hazards at the workplace. This is no different from other workers in the same job description in other parts of the world; but to varying degrees. This could result in a multitude of health outcomes, including NCDs. As an example, sedentary work, coupled with physical inactivity and unhealthy diets predispose to overweight and obesity.

The Integrated Health Screening and Health Promotion Programme among Civil Servants (IHSHP) 2007–2011, which was spearheaded by the Health Promotion Centre in Brunei, screened 21,437 civil servants from 12 government ministries. The population screened were females (55%), with varying age groups – 20–29 (15%), 30–39 (29%), 40–49 (35%), 50–59 (20%), and above 60 (1%). Results showed that 38% of civil servants were overweight and another 28% obese. Thirty-eight per cent of those screened had high blood pressure, 11% had high fasting blood glucose, and 25% high fasting blood cholesterol levels (6). The prevalence of self-reported stress was 49.5%, with females significantly more stressed (54.1%) compared to males (44.7%). Also, 23% of those screened reported not doing any exercise at all.

Data on specific parameters obtained from government OHS clinic activities also give cause for concern. In these OHS clinics, clients were exclusively from various government sectors which mandate a pre-employment medical fitness examination, as well as from private sectors requiring or requesting such assessment. These occupations include commercial airline staff, seafarers, construction, and mining workers. For certain jobs such as physicians and nurses in healthcare, police, fire and rescue, and aviation personnel, coverage for such examinations is 100%. For other sectors, such as manufacturing, periodic medical fitness to work examinations may be mandatory for high-risk jobs, but not required for low-risk jobs.

In 2012 (7), among approximately 3,500 clients assessed, 75% (compared to 68% in 2011) had undesirable BMI, 42% (compared to 39% in 2011) had elevated blood cholesterol levels, and 13% had raised blood pressure. Clients were referred to relevant specialties if further medical management was indicated. Workplace assessments were conducted when required, for example, workplace assessment of stress coupled with peer counselling and psychiatrist referral for a client with poor superior – employee relationship.

With efficient co-management of NCDs with primary healthcare doctors and other specialists, the OHS can serve as a gatekeeper for NCD screening at an earlier stage. This is not just for younger persons, for example, at the pre-employment stage, but also for older workers who are not concerned about their health status unless warranted by their employers through mandatory health screenings.

It is important that these screening programmes are not meant to be discriminatory against the employee. This applies equally to a prospective employee for certification of fitness for employment, or to someone in an existing job for termination or medical downgrading. Rather, the screening is meant for secondary prevention and also for the purpose of educating the employee and employer on the importance of health in the workplace. It also provides means and ways for an amicable win–win solution to be achieved should any adverse situation arise.

Thus, the OHS in Brunei Darussalam does not only issue fitness to work certificates, but is a significant partner in co-managing patients’ health conditions, with the advantage of further management at the workplace. The holistic approach of NCD management in the occupational setting is strengthened by employer and employee education and participation, targeting several approaches including risk management and advocating healthy lifestyles as part of a healthy workplace programme.

Several health guidelines and policies in Brunei Darussalam have been prepared with the involvement of the OHS (8). The OHS plays a pivotal role and contributes to the Ministry's and national vision and goal of addressing NCDs in alignment with the objectives of the Brunei National Multisectoral Action Plan for Prevention and Control of Non-Communicable Diseases (BruMAP-NCD) (8).

Nevertheless, challenges remain in providing a comprehensive OHS service to the working population. While Brunei Darussalam has a doctor and nurse density per 1,000 population of 1.1 and 6.1, respectively, which is among the highest in ASEAN (just behind Singapore and the Philippines) (9), occupational health is still considered a ‘unique specialty’ with less than 10 trained occupational health physicians in the government service at any one time. Coverage by the government service is estimated to be less than 15% of the entire working population. Furthermore, comprehensive data from OHS specifically pertaining to NCDs is very basic, with only available retrievable data on absolute numbers and percentage of clients within the job category assessed with specific NCDs.

The lack of availability of trained personnel and low coverage provided by the government sector is however partially offset by the presence of occupational health personnel and OHS in the private sector, for example, in the oil and gas industry, and appointed occupational health physicians in the private sector. Advocacy, increased awareness among all stakeholders, improved data collection, and management with opportunities for research as well as collaboration with relevant sectors will contribute to a more integrated approach in the efforts by OHS to address NCDs within the working population.

Despite these challenges, the government is committed in striving for better and ultimately optimal health for the Bruneian population, through various initiatives and policy directions such as the adoption of Health in All Policies approach. There is increased awareness from various sectors reflected by the rising demand for healthy workplace initiatives and programmes, following efforts in dissemination of statistics and dangers of NCDs and results of the IHSHP. Additionally, the OHS will continue to contribute to the ASEAN cause, such as through activities coordinated by Brunei Darussalam within the ASEAN Task Force on NCDs in the development of key indicators for healthy lifestyles and the monitoring and evaluation processes.

## Footnotes

1 Titah of His Majesty Sultan Haji Hassanal Bolkiah Mu'izzaddin Waddaulah ibni Al-Marhum Sultan Haji Omar ‘Ali Saifuddien Sa'adul Khairi Waddien, Sultan and Yang Di-Pertuan of Brunei Darussalam, on the occasion of the New Year's Day 2012, on the occasion of His Majesty's 66th Birthday 2012 and on the occasion of the Knowledge Convention (Majlis Ilmu) 2012.
